# Vitamin D and Its Role in Oral Diseases Development. Scoping Review

**DOI:** 10.3390/dj9110129

**Published:** 2021-11-02

**Authors:** Ekaterina Diachkova, Daria Trifonova, Elena Morozova, Gyuzel Runova, Igor Ashurko, Maria Ibadulaeva, Valentin Fadeev, Svetlana Tarasenko

**Affiliations:** 1 Department of Oral Surgery of the Institute of Dentistry, I.M. Sechenov First Moscow State Medical University (Sechenov University), 119048 Moscow, Russia; trifonova030897@mail.ru (D.T.); lemua@yandex.ru (E.M.); ashurko@yandex.ru (I.A.); prof_tarasenko@rambler.ru (S.T.); 2 Department of Fundamental Medical Disciplines, Medical Faculty, Moscow Region State University (MRSU), Str. Radio 10, Build. 1, 105005 Moscow, Russia; 3 Department of Endocrinology №1 of the Sklifosovsky Institute of Clinical Medicine, I.M. Sechenov First Moscow State Medical University (Sechenov University), 119048 Moscow, Russia; guzelvolkova@yandex.ru (G.R.); tmo-4509@mail.ru (M.I.); walfad@mail.ru (V.F.)

**Keywords:** vitamin D imbalance, osseointegration, dental implantation, maxillofacial region, oral diseases

## Abstract

Vitamin D is a fat-soluble secosteroid that plays a significant role in the whole body, including the maxillofacial region. The discovery of its receptors in many cells and organs made it possible to reveal the participation of vitamin D not only in the regulation of calcium phosphate metabolism, but also in immune processes, in providing anti-inflammatory and antimicrobial effects, slowing down cell proliferation and stimulating differentiation. In this literature review, we demonstrate the association between low vitamin D levels and the development of recurrent aphthous stomatitis, the course and response to treatment of squamous cell carcinoma of the oral cavity, the severity of periodontal diseases, and the processes of osseointegration and bone remodeling during dental implantation and guided tissue regeneration. The aim of our article was to demonstate a possible connection between vitamin D level and the oral diseases that can be presented at an oral surgery appointment, which will help clinicians to reduce the risk of early dental implant failure, ensure favorable outcomes of augmentative operations, as well as decrease the destructive effects of severe periodontitis and other conditions throug knowledge and timely lab tests and endocrinologist prescriptions.

## 1. Introduction

Vitamin D belongs to the group of fat-soluble secosteroid biomolecules. It is obtained in the body in two ways: alimentary (with food products and food additives intake) and through endogenous synthesis in the skin under UV radiation.

Worldwide vitamin D deficiency has increased interest in this compound, and therefore further study is warranted of its effect on various human organs and systems [1,2].

The most studied and effectively proven effects of vitamin D and its derivatives are the regulation of calcium phosphate metabolism and bone remodeling by enhancing intestinal absorption of calcium, increasing its reabsorption in the kidneys, and decreasing urinary secretion.

In addition, the discovery of vitamin D receptors in many cells and organs, for example, macrophages, monocytes, dendritic cells, cells of the placenta, the parathyroid gland and prostate, osteoblasts, smooth muscle cells and epithelial cells of the gingival attachment, contributed to the discovery of its “extra-osseous” effects [3,4]. A significant role of vitamin D has been proven in the immune processes, providing anti-inflammatory and antimicrobial effects, inhibiting cell proliferation, and stimulating differentiation.

The question of the effects of vitamin D on the oral cavity in modern dentistry remains poorly understood and requires further examination.

The aim of our article was the demonstration of a possible connection between vitamin D level and the appearance and course of different oral diseases that can be presented in oral surgeries.

## 2. Materials and Methods

The electronic search was performed via the PubMed database (www.ncbi.nlm.nih.gov/pubmed, accessed on 25 October 2021), Google Academy (https://scholar.google.ru/schhp?hl=ru, accessed on 25 October 2021) and the Cochrane Library, using a combination of keywords: “vitamin D deficiency”, “oral cavity”, “osseointegration”, “dental implant”, “periodontitis”. The articles from 2011–2021 were included. Relevant papers were included after manual searches within included articles. The journals on implantation and periodontics were manually examined, with the aim of searching for articles. The search was limited to clinical research articles and literature reviews describing the possible connection between vitamin D level and oral diseases that can often be presented at an oral surgery appointment. In vitro and animal studies were excluded because the results of animal experiments cannot always be generalized to humans [5,6].

## 3. Vitamin D Metabolism

There are two main native forms of vitamin D: vitamin D2 (ergocalciferol), which is contained in plants (yeast, mushrooms, crops) and enters the body only with food, and vitamin D_3_ (cholecalciferol), which is mostly synthesized in the skin from provitamin D3 (7-dehydrocholesterol) under the effects of sunlight. Another source of vitamin D_3_ is animals, such as wild salmon. Derivatives of vitamin D enter the extracellular matrix, and then bind to blood proteins in the bloodstream. Both forms are inactive and undergo further transformation in the body. Initially, hydroxylation occurs in the liver under the action of 25-hydroxylase transforming to 25(OH)D (calcidiol), which is the main circulating form of vitamin D. This indicator is used to quantify the serum level of vitamin D in clinical practice, since its half-life is up to 3 weeks [7]. According to the Russian Endocrine Society Clinical Practice Guideline, a level of vitamin D of 21–29 ng/mL (525–725 nmol/liter) is considered as insufficient, a 25(OH)D level below 20 ng/mL (50 nmol/liter) is defined as deficiency, and a blood level above 30 ng/mL is interpreted as optimal [2]. 

The subsequent stage of vitamin D metabolism, catalyzed by 1-hydroxylase, occurs mainly in the kidneys and, to a lesser extent, in bone tissue, lungs, liver, parothyroide glands and keratinocytes. The result of this process is the formation of the biologically active form of vitamin D, 1,25-dihydroxyvitamin D (1,25(OH)_2_D or calcitriol), which is responsible for all the effects [7,8,9].

## 4. Vitamin D Mechanism of Action

The mechanism of action of the active form of vitamin D is similar to that of other steroid hormones, and is realized by its binding to the nuclear receptor [10]. 1,25(OH)_2_D is a high-affinity ligand for the vitamin D receptor (VDR), which is present not only in the intestines, bone tissue and kidneys—main organs responsible for calcium phosphate metabolism—but also in more than 38 different target organs [11]. Its binding leads to the formation of a hormone–receptor complex that modifies gene expression by linking its specific domain with the regulatory DNA sequence [12]. Thus, there is activation of the synthesis of some proteins (for example, calcium-binding protein, osteocalcin, osteopontin) and inhibition of others (proinflammatory cytokines: IL-6, IL-8) [13,14].

The gene encoding the VDR is located in the chromosome 12 in position 12q13.1. The gene allele variations of VDR are relatively common in the population, with some differences between people of diverse ethnic groups. The polymorphism of the VDR gene may play a key role in the course of tumor progress, decreasing bone density, and increasing susceptibility to infections and autoimmune diseases, since it can influence the action of vitamin D on a cellular level, including calcium metabolism, transcription, cellular divisions and the initiation of the immunologic response [8,15,16].

A large number of studies have shown the correlation between low vitamin D levels and a number of different systemic diseases, i.e., diabetes mellitus, cardiovascular diseases (including coronary artery disease, congestive heart failure, valvular calcifications, stroke, arterial hypertension), autoimmune diseases (rheumatoid arthritis, systemic lupus erythematosus, multiple sclerosis, Crohn’s disease), chronic kidney disease, and many others [17].

Vitamin D’s regulation of calcium phosphate metabolism and bone remodeling, as well as its anti-inflammatory and immunomodulatory effects (regulation cell proliferation and differentiation), can significantly affect the health of the oral cavity [8,18,19,20,21]. A number of studies and reviews have demonstrated the association between low vitamin D levels and the course and frequency of recurrent aphthous stomatitis (RAS), the course and response to treatment of squamous cell carcinoma in the oral cavity, the severity of periodontal disease, and the processes of osseointegration and bone remodeling during dental implantation and guided tissue regeneration [18,22,23,24,25,26,27,28,29,30,31,32].

## 5. Oral Mucosa

### 5.1. Recurrent Aphthous Stomatitis (RAS)

Recurrent aphthous stomatitis (RAS) is a chronic mucosal disorder of the oral cavity, manifested in the presence of single painful erosions or ulcers of round or oval shape, with necrosis in the center and a hyperemia along the periphery. The etiology of this disease is still unknown, but dysregulation of the immune response is consider to be a risk factor, along with genetic defects, local trauma, emotional stress, and vitamin deficiency [18,33,34].

The significant role of vitamin D in the innate and acquired immune system, its ability to influence the synthesis of proinflammatory cytokines, and the presence of VDR on macrophages, dendritic cells, T- and B-lymphocytes, can explain the potential association with RAS [19,22,23].

According to a few studies [18,22,23,35], in patients with recurrent aphthous stomatitis, the level of serum 25(OH)D is significantly lower than in healthy people of similar ages and genders. Thus, Ainure Oztekin and Joshkun Oztekin [18] recommend vitamin D supplementation as a supportive treatment in patients with recurrent aphthous stomatitis. In the randomized clinical trial of Bakr Islam [36], a beneficial effect of topical oral vitamin D was demonstrated in its lowering of oral mucositis. However, in another study carried out by Krawiecka et al. [34], there was no significant difference in serum vitamin D levels (Table 1).

### 5.2. Cancer Malignancy of the Oral Cavity

One of the most common malignancies of the head and neck area is oral squamous cell carcinoma, with more than 300,000 new cases worldwide [37].

Molecular and cellular changes are associated with the influence of exogenous and endogenous factors (tobacco use or alcohol consumption, viral infections such as human papillomavirus (HPV), Epstein–Barr virus, hepatitis C virus, HIV) [38]. These multi-step processes contribute to the emergence of resistance to apoptosis in cancer stem cells, which prolongs their lifespans [24,39]. The disturbance of programmed cell death is a key factor in the carcinogenesis of squamous cell carcinoma of the oral cavity, and it manifests itself in a low response to radio- and chemotherapy as well as resistance to most anticancer drugs [15,39].

In this regard, considerable interest has arisen in examining the chemopreventive and therapeutic potential of vitamin D and its derivatives [40].

The antitumor activity of 1,25-(OH)_2_D_3_ in a number of cells is provided by its ability to induce apoptosis, and to inhibit invasion, cell proliferation, and tumor angiogenesis [15,39,41,42].

In cancer cells, 1,25-(OH)_2_D_3_ activates inhibitors of cyclin-dependent kinases (p21, p27) and mitogenic growth factors (IGF-1, EGF), and promotes the activation of TGF-β, thus exhibiting antiproliferative properties [43].

According to Udeabor S.E. et al. (2020) [24], more than 74% of patients with squamous cell carcinoma of the oral cavity showed a decrease in serum vitamin D levels compared with a control group with no history of cancer. A positive association between the risk of squamous cell carcinoma and vitamin D deficiency, especially at levels below 25 ng/ml, increases the likelihood of developing a malignant neoplasm by 1.65-fold [24].

Anand et al. concluded that patients with oral squamous cell carcinoma, who received vitamin D_3_ at a dose of 1000 International Units (IU) per day for 3 months, showed a reduction in the adverse effects associated with chemotherapy. There was a decrease in the severity of oral mucositis (a decrease in hyperemia, edema, ulceration, and pain), an improvement in swallowing function, and an increase in the quality of life compared with patients who did not receive vitamin D_3_ [15]. The same conclusion was reached by Mostafa et al. based on the results of their research [44]. 

**Table 1:** Literature analysis of vitamin D level and oral mucosa diseases connection.

Author, Year	Pathology	Patients, Age	Vitamin D Level	Diagnostics of Vitamin D Imbalance	Treatment of Vitamin D Imbalance	Vitamin D Level	Positive Results—Main Pathology	Probability
**RCT**								
Bakr SI et al., 2020 [36]	The effectiveness of topical oral vitamin D gel in prevention of radiation induced oral mucositis	45, Absent	<20 ng/mL	Vitamin D serum evaluation	Topical vitamin D oral gel, every 1 g of the gel contains 4000 IU. It was given twice daily.	Absent	Topical oral vitamin D gel has a beneficial effect in lowering oral mucositis development and in reducing pain sensation during the radiation period, especially when combined with conventional therapeutic agents.	*p* < 0.05
Lalla RV et al., 2012 [45]	Recurrent aphthous stomatitis	160, >18	Absent	Absent	A generic multivitamin supplement containing only the U.S. reference daily intake (RDI) of the essential vitamins A, B1, B2, B3, B5, B6, B9, B12, C, D and E.	Absent	Daily multivitamin supplementation, with the RDI of essential vitamins, did not result in a reduction in thenumber or duration of RAS episodes.	*p* = 0.69
**Others**								
Bahramian A et al., 2018 [22]	Recurrent aphthous stomatitis	52, 18–60	33.0.7 ± 12.41 ng/dL	The electrochemiluminescence technique	Absent	Absent	The serum levels of vitamin D in patients with RAS were significantly lower than those in healthy individuals.	*p* < 0.001
Öztekin A et al., 2018 [18]	Recurrent aphthous stomatitis	110, >18	11.00 ± 7.03 ng/mL	The electrochemiluminescence binding method (COBAS reagent kit; COBAS e601 analyzer series, Roche Diagnostics, Basel, Switzerland)	Absent	Absent	Lower vitamin D levels in patients with recurrent aphthous stomatitis compared to healthy controls.	*p* = 0.004
Khabbazi A et al., 2015 [23]	Recurrent aphthous stomatitis	95, 33.4 ± 9.8	12.1 ± 7.7 ng/dL	The Enzyme-Linked Immunosorbent Assay (ELISA) method	Absent	30 < 25(OH) D < 100 ng/dL	Insufficiency and deficiency of 25(OH) D in the RAS groups were more common than in the control group.	*p* = 0.0001
Udeabor SE et al., 2020 [24]	Oral squamous cell carcinoma (OSCC)	51, 59.33 ± 12.54	20.42 ± 12.02	Absent	Absent	>35 ng/mL	A positive association between vitamin D deficiency and OSCC risk.	*p* = 0.001
Krawiecka E et al., 2017 [34]	Recurrent aphthous stomatitis	66, 34.15 ± 12.26	16.81 ng/mL	The electro-chemiluminescence binding assay (ECLIA)	Absent	30–50 ng/mL	Vitamin D does not seem to be a trigger factor for RAS occurrence and does not appear to influence the severity of the disease in the studied group.	*p* = 0.2073
Grimm M et al., 2015 [39]	Oral squamous cell carcinoma (OSCC)	42, Absent	12.2 ng/mL	The radioimmunoassay at Biovis laboratory (Limburg-Offheim, Germany)	Absent	>35 ng/mL	A significantly increased expression of VDR was observed in tumor cells of OSCC.	*p* < 0.05
Anand A et al., 2017 [15]	Oral neoplasms	110, 42.67 ± 10.83	−1.90 ± 0.43; range −3 to 0	The chemiluminescent immunoassay method	1000 IU BD per day for 3 months	30–100 ng/mL	Vitamin D scores were significantly lower in cases compared to healthy controls.	*p* = 0.002
Zakeri M et al., 2021 [35]	Recurrent aphthous stomatitis	86, 15–40	13.19 ± 8.19 ng/mL	The enzyme-linked immunosorbent assay (ELISA), using a laboratory kit (Cat. No. EUROIMMUN, EQ. 6411–9601; PerkinElmer, Lübeck, Germany)	Absent	30–50 ng/mL	The serum levels of vitamin D are lower in patients with RAS in comparison with healthy controls.	*p* = 0.002
Mostafa B El-D et al., 2015 [44]	Head and neck squamous cell cancer	80, 54.8 ± 12.7	The median—40.35 ng/mL	The enzyme-linked immunosorbent assay (ELISA) technique using CALBIOTECH ELISA kit for Human VD3 Immunoassay (catalog no: VD220B; Calbiotech, Spring Valley, California, USA)	Absent	>80 nmol/l	Vitamin D deficiency is prominent in patients with head and neck squamous cell carcinoma before treatment compared to controls.	*p* < 0.001

## 6. Periodontal Diseases

In modern dentist’s practice, the problem of chronic generalized periodontitis is quite acute. This is due not only to the high prevalence of this pathology among the population—98%—but also to the lack of the expected effect of treatment [46].

Quite often, dentists are faced with resistance to the treatment of chronic generalized periodontitis, a decrease in the duration of stable remission and an increase in the aggressive course of periodontitis. The presented problems demonstrate the need for a more thorough study of the components of the pathogenesis of chronic generalized periodontitis, and the search for means of complex treatment [46].

Periodontitis is characterized by damage to the tissues surrounding the tooth, caused by the host’s immune inflammatory response to bacterial invasion. Since vitamin D plays an essential role in the metabolism of bone tissue and maintenance of the immune response, it is reasonable to suppose that its deficiency may affect the pathogenesis of the disease and the state of the periodontium [47,48].

The active metabolite of vitamin D—1,25(OH)_2_D_3_—is involved in specific immune defense and has an anti-inflammatory effect, acting on T- and B-lymphocytes, inhibiting the production of pro-inflammatory IL-6 and IL-8, which are involved in the development of acute inflammation [48,49,50,51].

The regulation of the nonspecific immune response occurs by stimulating the synthesis of antimicrobial peptides (defensins and cathelicidin) through vitamin D receptors (VDR), which have been found in monocytes, macrophages, neutrophils, and dendritic cells.

One of the defensins, beta-defensin 2, exhibits antimicrobial activity against oral pathogens, including bacteria associated with the development of periodontitis (Porphyromonas gingivalis, Fusobacterium nucleatum, and Aggregatibacter actinomycetemcomitans) [16,27].

The analysis of observations provided by Bashutski JD et al. [47] demonstrated that vitamin D deficiency leads to less effective outcomes after periodontal surgery (lower soft tissue attachment and changes in probing depth).

Pinto et al. [52] in their systematic review argued that the relation between periodontal disease and vitamin D deficiency may be justified, but most studies have significant limitations, which prevents the confirmation of the existence of this association.

Research by Isola et al. [28], as well as Anbarcioglu E et al. [27], showed that patients with periodontitis had lower serum vitamin D levels compared to healthy patients. Moreover, vitamin D deficiency negatively influenced the course of periodontal disease and increased the risk of aggressive periodontitis. Since this study confirms a relation between low serum vitamin D levels and the development of periodontitis, according to the authors, vitamin D assessment should be recommended at the beginning of periodontal therapy, as it may reduce the risk of developing this disease [28].

In addition to the above, the study by Garcia et al. demonstrates that calcium and vitamin D supplementation (1000 IU/day) had a moderate positive effect on periodontal health and improved clinical parameters [53]. The studies of Gao et al. and Meghil confirmed that supplementation with vitamin D significantly raised its serum level, improved such periodontal parameters as attachment loss and probing depth, and reduced systemic inflammation [54,55]. These results confirm the possibility of a positive effect of vitamin D on periodontal health (Table 2).

**Table 2:** Literature analysis of vitamin D level and periodontal diseases connection.

Author, Year	Pathology	Patients, Age	Vitamin D Level	Diagnostics of Vitamin D Imbalance	Treatment of Vitamin D Imbalance	Vitamin D Level	Positive Results—Main Pathology	Probability
**RCT**								
Gao W et al., 2020 [54]	Nonsurgical periodontal therapy	360, 30–70	Absent	Direct Elisa kit (Immunodiagnostic Systems Limited, IDS, East Boldon, Great Britain)	90 capsules of 2000 IU vitamin D3, 1000 IU vitamin D3 or placebo	Absent	Short-term supplementation of vitamin D significantly raised serum 25(OH)D levels and improved AL (attachment loss) and PD (probing depth) for moderate and deep pockets in patients with moderate to severe periodontitis	*p* < 0.05
Hiremath VP et al., 2013 [56]	Gingivitis	96, 18–64	20–65 ng/mL	Direct Elisa kit (Immunotek; Bensheim, Germany)	2000 IU/day, 1000 IU/day, 500 IU/day or placebo	Absent	Vitamin D has an anti-inflammatory effect in doses ranging from 500 to 2000 IU	*p* < 0.001
**Others**								
Meghil MM et al., 2019 [55]	Generalized chronic moderate to severe periodontitis	23, 44.8 ± 9.4	Non-deficient	Enzyme immunoassay (Immunodiagnostic Systems, Fountain Hills, AZ, USA)	4000 IU/day oral Vitamin D supplementation for 16 weeks	Absent	An important role for vitamin D supplementation in inducing local and systemic anti-inflammatory response and enhancing the autophagic profile in PD patients after scaling and root planning	*p* < 0.001
Bashutski JD et al., 2011 [47]	Periodontal surgery and teriparatide administration in patients with severe chronic periodontitis	40, 30–65	28% of enrolled participants presenting with mild deficiency (16–19 ng/mL), participants with moderate to severe deficiency were excluded	Serum vitamin D level	Daily 1000 mg calcium and 800 IU vitamin D oral supplements was initiated 3 days prior to surgery and continued for 6 weeks	20–74 ng/mL	It is advisable to ensure adequate vitamin D levels well in advance of periodontal surgery, to attain the best possible results	*p* < 0.01
Emrah A et al., 2018 [27]	The association between vitamin D concentration and both aggressive and chronic periodontitis	129, 21–47	11.22 ± 4.8 ng/mL—aggressive periodontitis 16.13 ± 8.3 ng/mL—chronic periodontitis	Liquid chromatography–mass spectrometry (LC-MS/MS) (München, Germany).	Absent	>20 ng/mL	The study showed that lower 25(OH)D concentrations were associated with a higher risk of aggressive periodontitis	*p* < 0.0002
Isola G et al., 2019 [28]	The association between serum vitamin D levels and periodontitis in patients with chronic periodontitis (CP) and coronary heart disease (CHD)	179, ≥18	CP—17.4 ± 5.2 ng/mL CP + CHD—16.5 ± 5.6 ng/mL	Serum vitamin D level	Absent	≥20 ng/mL	Patients with CP and CP + CHD presented significantly lower serum levels of vitamin D compared to CHD and healthy controls	*p* < 0.001
Laky M et al., 2019 [48]	The association between vitamin D concentration and periodontal diseases	58, 35.41 ± 7.7	Absent	An enzyme-immunoassay, EIASON 25-OH-VitaminD^®^ test kit, IASON GmbH, Graz, Austria	Absent	>30 ng/mL	25(OH)D deficiency is significantly associated with periodontal disease	*p* < 0.05
Agrawal AA et al., 2019 [50]	The association between the levels of vitamin D and calcium in the serum of periodontally healthy, chronic gingivitis and chronic periodontitis patients with and without type-2 diabetes mellitus	100, 30–50	chronic gingivitis—49.05 ng/mL chronic periodontitis—26.94 ng/mL	ELISA kit	Absent	Absent	Vitamin D and calcium levels are inversely correlated with random blood sugar and glycosylated hemoglobin, and, also probing pocket depth and clinical attachment loss, thus may contribute to increases in the severity of periodontal disease activity	*p* < 0.05
Garcia MN et al., 2011 [53]	Vitamin D and calcium supplementation for better periodontal health compared to patients not taking it	51, postmenopausal fem., males 50–80	Absent	Nutritional analysis	Vitamin D (400 IU/day) and calcium (1000 mg/day)	125 to 175 nmol/L	Calcium and vitamin D supplementation (1000 IU/day) had a modest positive effect on periodontal health	*p* = 0.058

## 7. Osseointegration

The most studied and proven effect of vitamin D is the regulation of calcium phosphate homeostasis and bone remodeling, which is realized through an increase in the intestinal absorption of calcium, its reabsorption in the renal tubules, the suppression of the synthesis and secretion of parathyroid hormone, the activation of osteoclasts, increases in the production of extracellular matrix by osteoblasts and the expression of genes of osteocalcin, osteopontin, calbindin and 24-hydroxylase [57,58,59,60,61,62]. Based on these data, vitamin D may play an essential role in bone regeneration during dental implantation and osteoplastic surgery (Table 3).

Today, implantology is developing with great pace, and dental implants have become a reliable way to rehabilitate missing teeth with functional and aesthetic results. The achievement of the long-term stable functioning of dental implants is ensured by their osseointegration, which is characterized by a direct strong connection between the bone and the implant surface in the absence of fibrous tissue. This phenomenon depends on many factors: the material, design and surface of the implant, the surgical technique and prosthodontic treatment protocol, as well as the quality of the bone and the regenerative capabilities of the body [63,64].

The process of the osseointegration of dental implants, which consists of several stages of bone remodeling, is accompanied by the active osteoclastic resorption of undifferentiated bone tissue [65,66]. Bone formation after osteoplastic operations occurs through the resorption of the graft and the replacement of the graft with the patient’s own bone tissue [25]. In these metabolic processes, osteoclasts play an active role along with osteoblasts [65]. 

Since the osseointegration of dental implants depends on the ability of bone to regenerate, it is assumed that the healing process and the formation of bone tissue around the implant are reduced with vitamin D deficiency [60,63,67,68,69].

Fretwurst et al. [57] demonstrated two clinical cases of early dental implant failure in patients with vitamin D deficiency. Both patients reported no systemic desease, were non-smokers, and took niether drugs nor alcohol. The clinical conditions were the same, so all implants were inserted with the torque < 35 Ncm, and primary stability was achieved. The surgical procedure was accompanied by a sufficient irrigation and surgical protocol. The results of the study show successful subsequent implant placement after vitamin D supplementation, and the restoration of its adequate level in the blood.

According to a case report of Bryce G and MacBeth N [26], a severe vitamin D deficiency in a patient who had undergone a single-stage implantation might have contributed to the absence of osseointegration five month post-operatively, and dental implant failure.

In observations reported by Schulze-Späte et al. [25], there is a significant correlation between increased serum vitamin D levels and the presence of osteoclasts around graft particles during augmentation. This may suggest a more pronounced metabolic activity, which promotes local remodeling in the augmentation area.

Moreover, Amr et al. [70] demonstrated the effect of vitamin D mixed with xenografts in alveolar bone augmentation. A statistically significant difference was observed in enhancing the bone formation and improving implant stability. 

The benefits of vitamin D were demonstrated in a study by Kwiatek J. et al. [71]. The bone level around the implant was significantly higher in patients who took vitamin D supplements, which allows us to suggest that this biomolecule is a potential factor that stimulates the remodeling process and the growth of bone tissue around the dental implant.

The level of crestal bone loss around the dental implant is one of the accepted methods for assessing its long-term survival. A significant correlation between the amount of marginal bone in patients with vitamin D deficiency who were supplemented with vitamin D and those who have not received it was observed in the study by Garg P et al. [62]. The authors concluded that normal levels of serum vitamin D can help to prevent the risk of early dental implant failure, enhance osseointegration and the healing process.

However, Javed et al. [60] and Boas et al. [72] suggested that the effect of vitamin D levels on implant osseointegration is still controversial.

Mangano et al. [59,73], in their retrospective clinical studies, noted a tendency towards an increased risk of early dental implant failure in patients with severe vitamin D deficiency. However, it was not possible to prove the relationship between these facts, since no statistically significant difference was demonstrated.

The success of a dental implant treatment is defined by both the achievement of osseointegration and peri-implant tissue health. Hence, Piccolotto A et al. [74] demonstrated differences in such parameters as the width of keratinized mucosa, bleeding index, probing depth, and the level of mesial and distal bone crest, depending on vitamin D status. According to this study, vitamin D supplementation for patients with deficiency has not manifested a statistically significant difference in clinical peri-implant tissue health, and the level of bone crest was classified as within the limits of the success criteria. However, it is worth taking into account that implant rehabilitation treatment had already been completed more than a year before the study, and vitamin D administration had been ongoing for 2 months.

According to some studies [73,75], the preoperative monitoring of serum 25(OH)D levels and, if necessary, the prescribing of oral vitamin D for several weeks before surgical intervention can accelerate the healing process and prevent early implant failure.

Vitamin D can influence osseointegration through the processes associated with healing and the marginal seals of soft tissues surrounding the implant and can help to reduce the development of bacterial infections and peri-implantitis [76,77]. The results of Acipinar S. et al.’s study [78] confirmed a significant decrease in serum 25(OH)D in a peri-implantitis group compared with a healthy control group.

**Table 3:** Literature analysis of vitamin D level and dental implantation connection.

Author, Year	Pathology	Patients, Age	Vitamin D Level	Diagnostics of Vitamin D Imbalance	Treatment of Vitamin D Imbalance	Vitamin D Level	Positive Results—Main Pathology	Probability
**RCT**								
Kwiatek J et al., 2021 [71]	Dental implantation	122, 43.8 ± 12.15	25.09 ± 11.04 ng/mL	ECLIA electrochemiluminescence on the Cobas 8000 (Roche) analyzer.	8000 IU/day	30–50 ng/mL	The correct level of vitamin D on the day of surgery and its deficiency treatment have a significant influence on the increase in the bone level at the implant site during the process of osseointegration.	*p* < 0.05
Schulze-Späte U et al., 2015 [25]	Maxillary sinus augmentation	20, 49.11 ± 12.24	Absent	Serum vitamin D level	5000 IU + 600 mg of calcium	The reference level was in accordance with manufacturer’s instructions	The increase in local bone-resorbing osteoclasts around graft particles and more pronounced bone remodeling.	*p* ≤ 0.05
**Others**								
Mangano FG et al., 2018 [73]	Early dental implant failure	885, 57.3 ± 14.4	29.5 ± 12.1 ng/mL	Serum vitamin D level	Absent	>30 ng/mL	There was a clear trend toward an increased incidence of early dental implant failure with a lowering of serum vitamin D levels, but no statistically significant difference.	*p* = 0.105
Mangano FG et al., 2016 [59]	Early dental implant failure	822, 57.3 ± 14.2	29.9 ± 12.1 ng/mL	Serum vitamin D level	Absent	>30 ng/mL	The incidence of early implant failures was higher in patients with low serum levels of vitamin D, but the difference between groups was not statistically significant.	Absent
Waskiewicz K et al., 2018 [75]	Oral surgery (tooth extraction, implantation, bone augmentation, orthognatic surgery)	46, 49.5	20.67 ± 11.536 ng/mL	Serum vitamin D level	Absent	30–80 ng/mL	A preoperative assessment including the dosage of vitamin D, promotes bone metabolism.	*p* = 0.322
Piccolotto A et al., 2019 [74]	Peri-implant tissues	33, 35–60	24.9 5 ± 0.96 ng/mL	Chemiluminescence in a clinical analysis laboratory	50,000 IU/week for 8 weeks	>30 ng/mL	There was no statistically significant difference in clinical peri-implant tissue health and the level of bone crest was classified as within the limits of success criteria.	*p* = 0.0034
Garg P et al., 2020 [62]	Dental implantation	32, 20–40	<30 ng/mL	Radioimmunoassay (RIA) method via fully automated RIA Analyzer SR300 by STRATEC Biomedical AG, Germany, using Beckman Coulter 25OH Vitamin D total RIA kit	60,000 IU/month for 3–6 months	30–100 ng/mL	It was observed that there is definitely much better osseointegration of implants in individuals supplemented with vitamin D.	Absent
Fretwurst T et al., 2016 [57]	Early dental implant failure	2, 48,51	11 ng/mL, 20 ng/mL	Serum vitamin D level	The dosage is not known	>30 ng/mL	Successful subsequent implantation in both patients.	Absent
Amr AEH et al., 2019 [70]	Alveolar ridge augmentation with simultaneously dental implantation	14, 28–40	Absent	Absent	Absent	Absent	Vitamin D enhanced the bone formation when mixed with xenografts in alveolar ridge augmentation and played a role in improving implant stability.	*p* ≤ 0.05

## 8. Medication-Related Osteonecrosis of the Jaw

Medication-related osteonecrosis of the jaw (MRONJ) is a condition of the oral cavity resulting in the exposure of underlying necrotic bone, persisting for more than 8 weeks, and it is a severe late complication of bisphosphonates, denosumab and antiangiogenics in the treatment of cancer-related conditions and osteoporosis [79]. Initially, only bisphosphonate-related osteonecrosis of the jaw (BRONJ) was described in the literature. However, in 2014, the American Association of Oral and Maxillofacial Surgeons recommended the change in the nomenclature of BRONJ to MRONJ, as it was found that other antiresorptives, such as denosumab, and antiangiogenic medications cause similar complications [80,81,82]. The pathophysiology of MRONJ is not completely understood, so its prevention and management remain challenging today.

Some of the risk factors strongly associated with MRONJ for patients undergoing antiresorptive therapy are infection and mechanical trauma after tooth extraction; moreover, some researchers have observed that a low vitamin D level also may be a risk factor for the development of MRONJ [80,83]. 

According to the study by Bedogni A. et al. [84], the hypothesis that vitamin D deficiency is more common in BRONJ+ than in BRONJ- patients is unlikely to be true. Even though the patients were matched for age and sex, and not all of them were supplemented with vitamin D, there are no significant differences in BRONJ+ and BRONJ- groups.

On the contrary, Demircan S. and Isler S.C. [85] demonstrated a significant association between vitamin D levels and MRONJ in their study. There was statistically lower vitamin D level than in the control group. The authors suggest that the clinician should follow up changes in biochemical markers of bone metabolism when managing oral surgery patients on bisphosphonates. However, there is a need for more studies of vitamin D levels, which will be evaluated separately.

A retrospective investigation by Heim N et al. [81] showed that the prevalence of MRONJ in patients treated with antiresorptive therapy seems to be increased by low serum vitamin D levels (Table 4).

MRONJ is a problem that can occur in all oral surgery. The most important factors that could prevent it are careful patient interviewing and the preparation of the patient for surgery, with the participation of an oncologist or endocrinologist, depending on the main disease. It is recommended to check the level of vitamin D and correct the supplementation depending on the results. Additionally, the clinician can use less traumatic surgical techniques, such as lasers.

**Table 4:** Literature analysis of vitamin D level and MRONJ connection.

Author, Year	Pathology	Patients, Age	Vitamin D Level	Diagnostics of Vitamin D Imbalance	Treatment of Vitamin D Imbalance	Vitamin D Level	Positive Results—Main Pathology	Probability
Heim N et al., 2017 [81]	Medication-related osteonecrosis of the jaw	63, 72.1 ± 10.73	20.49 ng/mL	Serum vitamin D level	Absent	>30 ng/mL	A significantly lower serum vitamin D level in subjects with stage 2 osteonecrosis than in patients without exposed bone.	*p* ≤ 0.05
Bedogni, A et al., 2019 [84]	Bisphosphonate-related osteonecrosis of the jaw	124, ≥18	Absent	Chemiluminescent immunoassay, Liaison, DiaSorin, Saluggia, Italy	Absent	>50 nmol/L	BRONJ+ and BRONJ– patients had the same frequency of vitamin D deficiency and most bone turnover markers.	Absent
Demircan S et al., 2020 [85]	Bisphosphonate-related osteonecrosis of the jaw	20, 56.89 ± 15.14	22.43 ± 9.36	High-performance liquid chromatography (HPLC) (ImmuChrom, Bensheim, Germany)	Absent	Absent	The vitamin D levels were statistically lower than in the control group, which can prove the strong relationship between changes in vitamin D levels and BRONJ	*p* = 0.046

## 9. Conclusions

The discovery of the “extra-osseous” effects of vitamin D has allowed us to pay attention to its significance in diseases of the maxillofacial region. The influence of vitamin D on immune processes has been discovered, along with its anti-inflammatory and antimicrobial effects. Decreasing cell proliferation and stimulating cell differentiation determines the development and course of RAS, squamous cell carcinoma of the oral cavity, periodontitis, and soft tissue healing after implantation and periodontal surgery.

Vitamin D is involved in the calcium phosphate metabolism of the bone tissue of the whole organism, and the maxillofacial region and has an active impact on the processes of osseointegration and bone remodeling. This knowledge can help clinicians to reduce the possible risk after different types of surgery, such as dental implantation and bone plasty, be more aware of complications, perform timely prophylaxis through the prompt prescription of lab tests, and include an appropriate specialist, such as an endocrinologist, in the doctors’ group. 

There are some works indicating the correlation between low serum levels of vitamin D and diseases of the maxillofacial region; to clarify the causal relationship, as well as to decide whether it is advisable to assess the status of vitamin D and the need to compensate for its deficiency, it is advisable to conduct further research. 

## Data Availability

Restrictions apply to the availability of these data. Data was obtained from other articles and are available from their authors or at articles’ URL with the permission of authors.
